# Demand for family planning among HIV positive women on ART: the case of South Gondar and North Wollo Zones Amhara region

**DOI:** 10.1186/s13104-016-1850-8

**Published:** 2016-01-25

**Authors:** Gedefaw Abeje, Achenef Motbaynor

**Affiliations:** School of Public Health, College of Medicine and Health Sciences, Bahir Dar University, P.O.Box 79, Bahir Dar, Ethiopia; College of Health Sciences, Madawalabu University, Bale Goba, Ethiopia

**Keywords:** Amhara, Demand for family planning, ART, Unmet need, South Gondar, North Wollo

## Abstract

**Background:**

Although family planning for human immune deficiency virus positive women has numerous advantages, evidences in different parts of the world showed the existence of persistent unmet need. There were few studies done in Ethiopia on level of unmet need for family planning among women in reproductive age on antiretroviral therapy (ART). This study was therefore done to determine the level of demand and unmet need for family planning among women on ART in South Gondar and North Wollo Zones, Amhara region.

**Methods:**

Institution based cross-sectional study design was used. Data was collected from June 15 to 25, 2013 in South Gondar and North Wollo Zones. Study participants were recruited from six health centers and two hospitals. The study participants were proportionally allocated to the health institutions. Multistage sampling technique was used to recruit study participants. Trained nurses interviewed the respondents using pretested structured Amharic questionnaire. Data was entered, cleaned and analyzed using Statistical Package for Social Science version 16. Ratios and proportions were computed to determine demand and unmet need for family planning.

**Results:**

A total of 530 women in reproductive age on ART were interviewed in this study. Two hundred ninety-three women were married. Fourteen (2.6 %) women were pregnant at the time of interview. Five of these pregnancies were not planned. In this study, 242 (45.7 %) women reported that they were using contraceptives. Most women (74.4 %) were using injectable (depo). Among those who were not using contraceptives, 84 (29.2 %) reported that they will use in the future. Fifty-two (61.9 %) of them said that they will use injectables (depo). In this study, the total demand for family planning among women on ART was 86.7 %. From this, 62.1 % and 24.6 % was met and unmet need respectively.

**Conclusions:**

This study revealed that the level of demand and met need for modern contraceptives among reproductive age women on antiretroviral therapy in South Gondar and North Wollo Zones was higher than that of sexually active married women in Ethiopia. But the level of unmet need is still similar with that of sexually active married women in Ethiopia.

## Background

There were 2.7 million new human immune deficiency virus (HIV) infections in 2010, including an estimated 390,000 children globally [1]. Sub-Saharan Africa remains the region most heavily affected by HIV. Ethiopia was also one of the Sub-Saharan countries affected by the epidemic, heterosexual HIV transmission being the main route followed by mother to child transmission [2].Women of reproductive age account for 60 percent of all adult HIV infections [3].

Preventing unintended pregnancies among women living with HIV is one of the four comprehensive approaches that the World Health Organization (WHO) promotes to prevent the transmission of HIV from mother to baby [1]. Family planning reduces HIV positive women’s vulnerability to morbidity and mortality related to pregnancy and lactation [4–7]. Providing family planning services for HIV positive clients is also cost effective [8, 9].

All available family planning methods such as oral contraceptives, injectables, implants, intrauterine device (IUD), barrier methods and spermicides can be appropriate choices for all HIV positive women including those who do not have advanced disease and on effective antiretroviral (ARV) treatment [10, 11].

Unmet need for family planning is high in Sub-Saharan Africa. A study done in Eastern Sudan on July 2012 revealed that 44.8 % of ever married woman in reproductive age had unmet need for family planning [12]. The 2011 Ethiopian Demographic and Health Survey (EDHS) reported that unmet need for modern contraceptives among currently married women in Ethiopia was 25 %. The report added that injectables were the most popular method used by 21 % of currently married women [13].

There were some evidences that showed persistence of high “unmet need” for FP among HIV positive patients. A study in Uganda by the Centers for Disease Control (CDC) and Prevention showed that pregnancies were occurring among ART users who did not want any more children [14].

A study done among HIV positive women on antiretroviral therapy in Johannesburg showed that nearly one out of three women had unmet need for family planning [15]. Another study in South-western Uganda to assess contraceptive use and associated factors among women enrolled in HIV care indicated that the proportion of women using contraceptive was 27.8 %. In this study, the most common method used was injectable hormones (51.7 %), followed by condoms (29.6 %), and oral contraceptives (8.7 %) [16].

A cross sectional study design to assess demand for family planning among women voluntary counselling and testing (VCT) clients in Dessie town, Northeast Ethiopia showed that the total demand for family planning among sexually active women VCT clients was 86 %. This study added that HIV positive VCT clients have high (62 %) unmet need for family planning than HIV negatives (53 %) [17]. A study in Lesotho showed that unmet need for contraception was lower among HIV positive women (21.6 %) compared to HIV negative women (32.5 %) [18].

It was important to identify existing and anticipated needs for family planning among women on ART. Information on current and projected levels and structure of demand for family planning is always crucial to program planning and for demand generation. But there were few evidences on level and demand for family planning among women on ART in Ethiopia. Therefore, this study was done to determine the level of demand and unmet need for family planning among women on ART in South Gondar and North Wollo Zones, Amhara region, Ethiopia. Demand for family planning in this study referred to the number or proportion of women married or in union at the time of the study who were fecund and who desired to either terminate childbearing or to postpone their next birth for 2 years from the time of survey. This includes those women who were using contraceptives and those who wished to use but were not using now [19].

## Methods

Institution based cross sectional study design was used to determine demand for family planning among women in reproductive age on ART. A multistage sampling technique was used to select the study participants. Data was collected from two selected hospitals and six health Centers of South Gondar and North Wollo zones from June 15 to 25, 2013. Twenty-five health Centers and three hospitals provide ART services in these zones (Amhara region health bureau, 2012 annual report, unpublished). From these, three health centers and one hospital from each zone were selected randomly. ART service users of the previous month were identified in each health institution. Finally total sample size was proportionally allocated to each health institution based on the number of reproductive age women who were receiving ART on each health institution.

Data collectors first identify three reproductive age women who came for ART service and select one of them as study participant. After that, they included every third reproductive age women who came for ART service. Sample size was calculated using single population proportion formula. Female nurses at each health institution collected the data using structured and pretested Amharic (the local language) questionnaire. Onsite training was given for data collectors on the objective of the research, on the procedure of data collection and interview techniques. Pre test was done. The supervisors checked the collected data daily. Incomplete questionnaires were returned back to data collectors for correction. Data was cleaned, entered and analyzed using SPSS 16. Ratios and proportions were computed to determine demand and unmet need for family planning.

Ethical approval was obtained from Amhara region health bureau. Permission to conduct the study was obtained from zonal, district and health center administrators. Informed consent was obtained from each study participant. To ensure confidentiality names of the study participants were not recorded on the questionnaire. In addition, the collected data was kept locked in file cabinet. Information about each contraceptive method was given for those who had misconception or misunderstanding about contraceptives and were linked to family planning unit of the health institutions for further counseling.

## Results

A total of 530 women in reproductive age on ART were enrolled in this study. Their mean age was 32.1 years with standard deviation of 6.4 years. Two hundred ninety-three of the women were married. Majority of the respondents were orthodox Christian. Almost all (99.6 %) were Amhara. Two hundred thirty-four respondents reported that they have attended formal education but about 66 % attended only primary level education (Table 1).

**Table 1:** Socio-demographic characteristics of women in reproductive age on ART in South Gondar and North Wollo Zones, June 2013

Socio-demographic characteristics	Number (%)
Marital status (n = 530)	
Single	53 (10.0)
Married	293 (55.3)
Divorced	119 (22.5)
Widowed	65 (12.5)
Residence (n = 530)	
Rural	149 (28.1)
Urban	381 (71.9)
Religion (n = 530)	
Orthodox Christian	442 (83.4)
Muslim	87 (16.4)
Protestant	1 (0.2)
Formal education	
Yes	234 (44.2)
No	296 (55.8)
Level of education completed (n = 234)	
Primary (1-8)	154 (65.8)
Secondary (9-12)and TVET	79 (33.8)
Above secondary	1 (0.4)
Currently employed (n = 530)	
Yes	42 (7.9)
No	488 (92.1)

Two hundred thirty-four (61.1 %) respondents reported that they had sex within the past 12 months. From all women who have history of pregnancy, 43 % had one or two pregnancies whereas 72 (15.6 %) reported more than 4 pregnancies. Twenty-four percent of women included in the survey had history of abortion. From these, about 29 % reported two or more abortions. Fourteen (2.6 %) women reported that they were pregnant at the time of interview. Five of these women reported that their pregnancy was not planned (Table 2).

**Table 2:** Obstetric history of women reproductive age on ART in South Gondar and North Wollo zones, June 2013

Variable	Number (%)
History of pregnancy	
Yes	461 (87.0)
No	69 (13.0)
Number of pregnancies	
1–2	198 (43.0)
3–4	191 (41.4)
More than 4	72 (15.6)
History of abortion	
Yes	127 (24.0)
No	403 (76.0)
Number of abortion	
1	90 (70.9)
2 or more	37 (29.1)
Alive child/children	
Yes	434 (81.9)
No	96 (18.1)
Number of live children	
Less than 4	344 (79.3)
4 or more	90 (20.7)
Currently pregnant	
Yes	14 (2.6)
No	516 (97.4)

Five hundred fourteen (97 %) women reported that they heard about modern contraceptives. About 93 % of women heard this information from health professionals (Table 3). Women were also asked if they were counseled about family planning. About 88 % of them reported that they were counseled within the last 12 months. Most of them (99 %) agreed that the counseling they received was adequate.

**Table 3:** Information about contraceptive for women on ART in the last 12 months, North Wollo and South Gondar Zones, June 2013

Question	Number (%)
Heard about modern contraceptives in the last 12 months (n = 530)	
Yes	514 (97.0)
No	16 (3.0)
Type of contraceptive heard (n = 514)	
Pills	356 (69.3)
Depo	418 (93.6)
Condom	213 (41.4)
Implants	130 (25.3)
IUCD	101 (19.6)
Tubal ligation	25 (4.9)
Vasectomy	22 (4.3)
Source of information for modern contraceptives	
Health professionals	479 (93.2)
Television	129 (25.1)
Radio	103 (20.0)
Neighbors	75 (14.6)
Counseled about family planning in the last 12 months (n = 530)	
Yes	469 (88.5)
No	61 (11.5)
The counseling is sufficient (n = 469)	
Yes	465 (99.1)
No	4 (0.9)

In this study, 242 (45.7 %) women reported that they were using different methods of contraceptives. The contraceptives used at the time of interview were injectibles (depo) (74.4 %), condom (10.3 %), pills (7.4 %), IUCD (4.5 %) and implants (3.7 %). Among women who were not using contraceptives, 84 (29.2) reported that they will use contraceptives in the future. Fifty-two (61.9 %) of them said that they will use injectables (depo). This study revealed that the total demand for family planning among women on ART in South Gondar and North Wollo zones was 86.7 %. From this, 62.1 % was met need and 24.6 % was unmet need.

## Discussion

This study showed that most women (97 %) included in the survey had heard about modern contraceptives. About 93 % women heard this information from health professionals. This finding was similar with 2011 EDHS report [13]. This may suggest that women on ART were exposed to the same level of information about family planning as the general population. About 88 % of women reported that they were counseled about family planning. Most importantly, almost all of them agreed that the counseling they received was adequate. This is good as women on ART may have many concerns about contraceptives.

In this study, 242 (45.7 %) women reported that they were using different methods of contraceptives which was higher than the 2011 EDHS report [13]. The reason for this difference is that women on ART have more demand on family planning than the general population. The proportion of women using contraceptives in this study was also higher than a study in Southwestern Uganda [16]. The reason for this difference was that women in this study were on ART which may increase their demand for family planning. Injectable (depo) was the contraceptive method used by most of the women (74.4 %). This finding was similar with a study among HIV positive women in Southwestern Uganda [16]. This was also the contraceptive most frequently used by other women in Ethiopia [13]. Among twomen who were not using contraceptives, 84 (29.2 %) reported that they will use contraceptives in the future. Fifty two (61.9 %) of them said that they will use injectables (depo).

This study revealed 86.7 % total demand for family planning among women on ART in South Gondar and North Wollo Zones. From this, 62.1 % was met need and 24.6 % was unmet need. This demand was higher than demand for family planning among all married women in Ethiopia (52 %) but comparable with study among sexually active women VCT clients (86 %) in Dessie town [13, 17]. The level of met need is higher but unmet need is comparable with the EDHS 2011 finding [13].

The unmet need for family planning in this study (24.57 %) was lower than studies conducted in Johannesburg among HIV positive women on antiretroviral therapy [15] and study in Dessie town among HIV positive women VCT clients [17] but slightly higher than a study done in Lesotho [18]. The explanation for this difference may be the difference in the study design in case of the Johannesburg’s study and the difference in the study population in case of Dessie town.

This study addressed one of the key areas which was important to assist the effort needed to prevent mother to child transmission of HIV/AIDS. It had showed the current level of demand for family planning which is important for program planning. The limitation of this study was that it was not able to identify factors associated with unmet need since the study participants were homogenous in most of the variables.

## Conclusion

**Fig. 1:**
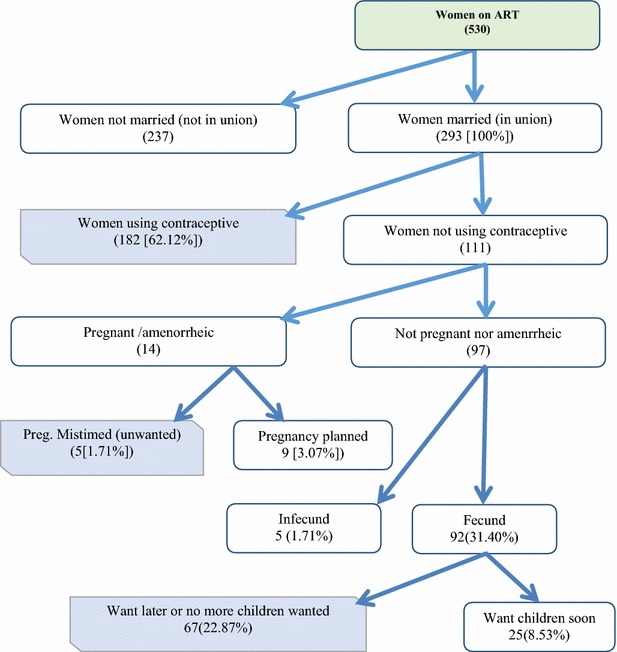
Demand for family planning among women on ART in South Gondar and North Wollo Zones, June 2013

This study revealed that the level of demand and met need for modern contraceptives among reproductive age women on ART in South Gondar and North Wollo Zones was higher than that of sexually active married women in Ethiopia. Injectables (depo) was the method used by most of the users and it was the one intended to be used by those who were not using at the time of interview but planned to use in the future Fig. 1.

All heath institutions should ensure the availability of injectables (depo) in their stock since it was the preferred method by most users of modern contraceptives. It was also the method that most women in this study intended to use. It is also important to conduct further research to identify the reason for unmet need for family planning among women on ART with large sample size.

## Abbreviations

AIDS: acquired immune deficiency syndrome
ART: antiretroviral therapy
ARV: antiretroviral
CDC: Centers for Disease Control
EDHS: Ethiopian Demographic and Health Survey
HIV: human immune deficiency virus
IUCD: intrauterine contraceptive device
IUD: intrauterine device
SPSS: Statistical Package for Social Science
USAID: United States Agency for International Development
VCT: voluntary counselling and testing
WHO: World Health Organization
